# Short Duplex Module Coupled to G-Quadruplexes Increases Fluorescence of Synthetic GFP Chromophore Analogues

**DOI:** 10.3390/s20030915

**Published:** 2020-02-09

**Authors:** Snizhana O. Zaitseva, Nadezhda S. Baleeva, Timofei S. Zatsepin, Ivan N. Myasnyanko, Anton V. Turaev, Galina E. Pozmogova, Alexei A. Khrulev, Anna M. Varizhuk, Mikhail S. Baranov, Andrey V. Aralov

**Affiliations:** 1 Shemyakin-Ovchinnikov Institute of Bioorganic Chemistry, Moscow 117997, Russia; snezaitseva@gmail.com (S.O.Z.); nsbaleeva@gmail.com (N.S.B.); conzbutcher@gmail.com (I.N.M.); gluttony5@gmail.com (A.A.K.); 2 Skolkovo Institute of Science and Technology, Moscow 121205, Russia; tsz@yandex.ru; 3 Department of Chemistry, Lomonosov Moscow State University, Moscow 119992, Russia; 4 Federal Research and Clinical Center of Physical-Chemical Medicine, Moscow 119435, Russia; stepanishchev@phystech.edu (A.V.T.); pozmge@gmail.com (G.E.P.); aliviense@gmail.com (A.M.V.); 5 Moscow Institute of Physics and Technology, Dolgoprudny 141700, Russia; 6 Center for Precision Genome Editing and Genetic Technologies for Biomedicine, Moscow 119435, Russia; 7 Pirogov Russian National Research Medical University, Moscow 117997, Russia

**Keywords:** green fluorescent protein (GFP) chromophore, fluorogenic dye, G-quadruplex, quadruplex-duplex junction, aptamer, biosensors

## Abstract

Aptasensors became popular instruments in bioanalytical chemistry and molecular biology. To increase specificity, perspective signaling elements in aptasensors can be separated into a G-quadruplex (G4) part and a free fluorescent dye that lights up upon binding to the G4 part. However, current systems are limited by relatively low enhancement of fluorescence upon dye binding. Here, we added duplex modules to G4 structures, which supposedly cause the formation of a dye-binding cavity between two modules. Screening of multiple synthetic GFP chromophore analogues and variation of the duplex module resulted in the selection of dyes that light up after complex formation with two-module structures and their RNA analogues by up to 20 times compared to parent G4s. We demonstrated that the short duplex part in TBA25 is preferable for fluorescence light up in comparison to parent TBA15 molecule as well as TBA31 and TBA63 stabilized by longer duplexes. Duplex part of TBA25 may be partially unfolded and has reduced rigidity, which might facilitate optimal dye positioning in the joint between G4 and the duplex. We demonstrated dye enhancement after binding to modified TBA, LTR-III, and Tel23a G4 structures and propose that such architecture of short duplex-G4 signaling elements will enforce the development of improved aptasensors.

## 1. Introduction

Aptamers are DNA or RNA fragments that recognize their targets with high sensitivity and selectivity due to their unique three-dimensional structures that can be tuned using systematic evolution of ligands by exponential enrichment (SELEX) [1,2,3,4,5]. They have several advantages over antibodies, such as high stability, lack of immunogenicity, low molecular weight, simplicity of synthesis and chemical modification or conjugation. Aptamers are widely used as a recognizing part of biosensors in the detection of various analytes using colorimetry, fluorescence, Raman spectroscopy, electrochemistry, acoustic, and heat-transfer methods [6,7,8,9,10,11,12]. Biosensors that use fluorescence to generate the output signal represent non-destructive and non-invasive molecular tools with rapid response, high sensitivity and high temporal/spatial resolution [13,14]. However, most of them still require the introduction of fluorophores and/or quenchers, which increase the cost of biosensors and could even deteriorate the recognition properties of aptamers. To exclude chemical or enzymatic labelling, aptamer-based elements have been proposed that can selectively bind to fluorogenic dyes that increase fluorescence after binding to them [15]. These biosensors contain dye-binding “signaling” and analyte-binding “recognizing” elements typically fused via a short stem linker in such a way that the analyte binding provokes reorganization of the “signaling” element and binding to a dye resulting in the fluorescence increase. Such RNA-based signaling elements are present in malachite green/RNA aptamer pair [15,16] and GFP chromophore analogue DFHBI/split spinach aptamer pair [17,18]. Despite increased chemical and biological stability of DNA aptamers compared to RNA ones, the only example of such a biosensor based on light-up dye–DNA aptamer pair as “signaling element” is dapoxyl dye/DAP-10-42 for thrombin and ATP detection [19] and “split” DAP-10 variant for nucleic acids analysis [20]. Several examples are devoted to label-free sensors utilizing G-quadruplex DNAs and their light-up ligands as “signaling elements”, including zinc(II)-protoporphyrin IX [21], thioflavin T [22], iridium(III) complexes [23] and crystal violet [24]. However, the enhancement of fluorescence in these systems is relatively weak, and most of these molecules can increase fluorescence by nonspecific binding to different sequences of G-quadruplexes, which leads to false-positive signals. In this regard, development of novel dye-G4 pairs with increased selectivity and improved photophysical properties is of unmet need.

**Figure 1:**
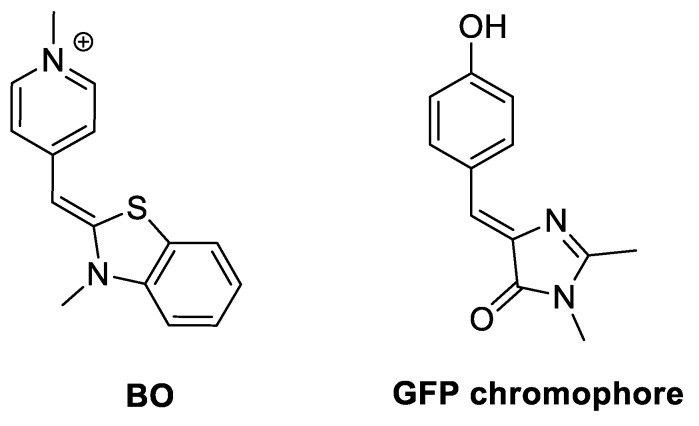
Structures of **BO** and GFP chromophore.

Anti-thrombin aptamer (TBA15) that forms an antiparallel two-tetrad G-quadruplex is among the most well-known and studied aptamers. TBA15 recognizes the fibrinogen-binding exosite responsible for binding to fibrinogen and cellular protease-activated receptors (PAR). As a result, it inhibits the thrombin-catalyzed conversion of fibrinogen into fibrin and thrombin-induced platelet aggregation and does not directly modulate the enzyme active center [25,26]. TBA15 as a drug for the prevention of thrombosis failed in clinical trials due to suboptimal dosing profiles in humans. In this regard, a large number of improved TBA15 analogues were developed, including hybrid analogues TBA31, RE31, and NU172 consisting of duplex/quadruplex modules and RA36 formed by two quadruplexes [27,28,29,30,31]. Aptamers RE31 and NU172 adopt the structure where two modules are perfectly stacked on the top of each other, firmly connected by a well-structured junction. Similar to RE31, TBA31 also contains a TBA15 module flanked by two partly complementary strands and also appears to fold into the structure with an inter-module junction. Recently, we found that the cyanine dye, benzothiazole orange (**BO**, Figure 1), exhibits strong enhancement in fluorescence quantum yield after binding to TBA31, and this fact was attributed to dye interactions with the junction between two modules [30]. Molecular dynamics (MD) simulations revealed that **BO** could be positioned in the minor cavity and fixed in a planar conformation between the G24-A8 pseudo-pair and the outer G-quartet. Thus, two-module structure with the inter-module junction makes TBA31 an excellent candidate to study the effect of attached duplex modules on the increase of dye fluorescence upon binding to G4. Such pairs of fluorogenic dyes and anti-thrombin aptamers can be used as signaling elements in the design of dual-element sensors.

Inspired by some structural similarity of **BO** and GFP chromophore (Figure 1) and also to provide a diversity of dye structures, we studied 83 GFP chromophore analogues. We also varied the length of the duplex module to find out if duplex stability can influence on the light-up of fluorescence. As a result, we found several dyes that increase fluorescence up to 20X when bounded with two-module TBA aptamers in comparison to parent TBA15. Finally, fluorescence parameters of GFP analogues in complex with other G4 structures containing additional stabilizing module (duplex for LTR-III or triple capping for Tel32a) were investigated.

## 2. Materials and Methods

### 2.1. Synthesis of BO and GFP Chromophore Analogues

All compounds were synthesized as previously described (see references in Table S1). All fluorophores were dissolved in DMSO (Sigma Aldrich, “for molecular biology” grade, #cat D8418) in 5 mM concentration and stored in dark place at −20 °C less than 3 months.

### 2.2. Oligonucleotides

Oligonucleotides (Table 1) were purchased from Evrogen, Russia (purity ≥ 95% by HPLC). For all experiments with oligoribonucleotides, DEPC-treated water was used.

### 2.3. CD Spectra and Melting Experiments

Circular dichroism (CD) spectra and melting curves of the oligonucleotides were recorded using a Chirascan spectrophotometer (Applied Photophysics, UK), equipped with a thermostated cuvette holder. Oligonucleotides were dissolved in a buffer (20 mM Tris-HCl, 100 mM KCl pH 7.8) to final concentrations of 2.5–5 μM. Prior to the experiments, the samples were denatured at 95 °C for 5 min and snap-cooled on ice to ensure intramolecular G4 folding. The control duplex was denatured at 95 °C for 5 min and cooled slowly to room temperature. CD spectra were recorded at 5 °C. Quartz cuvettes with an optical path of 1 cm were used for both CD and absorbance measurements. In the melting experiments, CD or absorbance were registered every 1 °C over the range of 5–95 °C. The heating rates were 1 °C/min. Melting temperatures were determined from the maxima of the first derivatives of the melting curves.

### 2.4. Measurement of Fluorescence and Absorption Spectra

Photophysical properties of unbound fluorophores were investigated using 5 µM solutions in water at room temperature on a Varian Cary 100 spectrophotometer and Agilent Cary Eclipse fluorescence spectrophotometer.

### 2.5. Primary and Secondary screening of the fluorophore library

Fluorophore binding to oligonucleotides was studied in Tecan Infinite 200 Pro M Nano reader. Solutions contained 5 µM of oligonucleotide and fluorophore in 20 mM Tris-HCl, pH 7.8, 100 mM KCl buffer. Fluorescence intensity enhancement was defined as the ratio of fluorescence intensity of solution of the fluorophore together with the oligonucleotide to the fluorescence intensity of solution of the free fluorophore upon excitation at 380, 430, 480, 530 и 580 nm. The experiments were carried out in triplicates (secondary screening) for the leader dyes (Table 2 and Table S1) and in singlicate (primary screening) for the remaining fluorophores (Table S1). Fluorescence spectra of the leader compounds (free and in complex with TBA15 and TBA31) were additionally recorded in the same buffer on Agilent Cary Eclipse fluorescence spectrophotometer.

## 3. Results and discussion

### 3.1. Interaction of GFP Chromophore Analogues with TBA15 and TBA31

**Figure 2:**
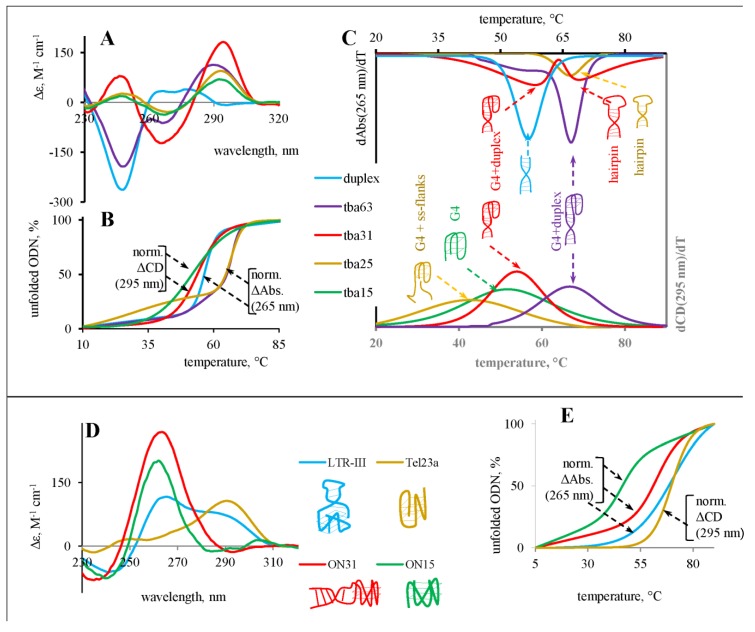
Secondary structures of the ONs: CD spectra and melting curves. (**A**) CD spectra of the deoxy-TBA ONs. (**B**) Melting curves of the deoxy-TBA ONs. (**C**) First derivatives of the deoxy-TBA melting curves and schematic representations of the ON secondary structures. (**A**–**C**) have a joint legend. (**D**) CD spectra of the ribo-ONs (ON31 and ON15), LTR-III and Tel23a and schematic representations of the possible ON secondary structures. (**E**) Melting curves of the ribo-ONs, LTR-III and Tel23a. (**D**) and (**E**) also have a joint legend. CD spectra were recorded at 5°C. ON concentrations were 2.5 uM (TBA63, TBA31, and ON31), 3.5 uM (TBA25 and LTR-III), or 5 uM (TBA15, Tel23a, and ON15). ΔCD/Abs changes were normalized (divided by Δmax.) to calculate unfolded ON fraction.

Anti-thrombin aptamer TBA15 forms an antiparallel two-tetrad G-quadruplex and TBA31 is a TBA15 analogue with an additional duplex module. First, we confirmed that both structures were folded correctly under conditions used in the study. Indeed, according to CD experiments, characteristic peaks at 295 nm (positive) and 265 nm (negative) confirmed the presence of the antiparallel G4 structure, while negative peaks at 240 nm indicated the presence of a duplex fragment for TBA31 (Figure 2A). To characterize thermal stabilities, we performed melting experiments (Figure 2B,C, Table 1). Monitoring changes in CD 295 nm (the antiparallel G4-specific maximum) resulted in monophasic melting curves for TBA 15 and TBA 31. At the same time, monitoring absorbance at 265 nm resulted in a biphasic curve for TBA31. We attribute the major inflection, which is observed in both CD (295 nm) and Abs (265 nm) curves, to melting of the double-module (G4+duplex) structure. The second (minor) inflection in the Abs. (265 nm) curve can be attributed to the admixture of a hairpin structure (see red schemes in Figure 2C).

**Figure 3:**
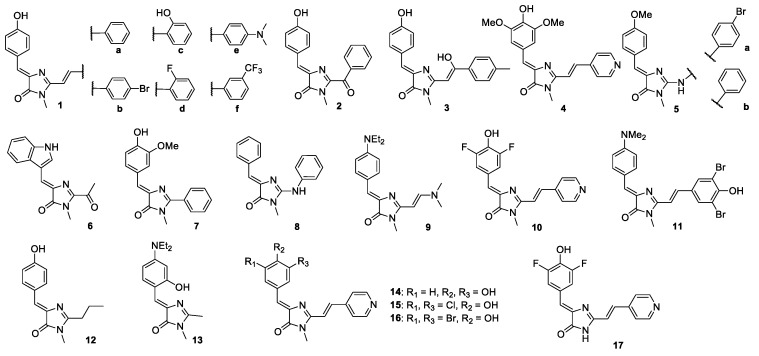
Structures of GFP fluorophore analogues used in the study.

Then, we studied the fluorescence enhancement of GFP chromophore analogues after mixing with an equimolar amount of the corresponding oligonucleotide (Table S1). Among 83 compounds tested, 14 showed sensitivity to the presence of an additional duplex module (Figure 3, Table 2). Thus, interaction with “naked” G4 (TBA15) led only to 2-3-fold fluorescence enhancements, whereas mixing with two-module (duplex+G4) TBA31 gave a 7-20-fold increase (Figure S1). The relationship between the structure and the ability of fluorophores to light up remains unclear, although leader compounds **1a–e**, **2** and **3** share a common 4-(4-hydroxybenzylidene)-1-methyl-1H-imidazol-5(4H)-one fragment typical for the parent GFP chromophore. In addition, derivative **6** with the highest light-up effect consists of conjugated bicyclic and monocyclic aromatic rings - a structure that has much in common with the **BO** structure [32]. It should be noted that developed compounds have emission maxima in a wide wavelength range (450–616 nm), which allows multiplex detection together with alternative approaches. Thus, we found several leaders compounds suitable for further studies.

### 3.2. Interaction of GFP Chromophore Analogues with Truncated (TBA25) and Elongated (TBA63) Versions of TBA31

To study the effect of the duplex module length/stability on the fluorophore ability to increase fluorescence intensity due to accommodation in the inter-module cavity, truncated (TBA25) and elongated (TBA63) versions of TBA31 were synthesized. In addition, a control duplex ON1-ON2 - a double-stranded part of TBA63 was prepared. Again, secondary structures of the oligonucleotides were confirmed by CD spectroscopy (Figure 2A). The spectrum of the duplex ON1-ON2 contained a large negative peak at 247 nm and a broad positive peak at 265–280 nm, which is typical for AT-rich duplexes. Monitoring by CD at 295 nm gave monophasic melting curves for TBA25 and TBA63, while at 265 nm a seemingly monophasic melting curve for TBA25 and a biphasic curve for TBA63 were obtained (Figure 2B,C, Table 1). In most cases, a decrease in the duplex module length led to an increase in the fluorescence intensity of fluorogenic dye in the complex (see TBA25 column in Table 2). This effect could be explained by the reduced rigidity of the two-module structure, which might facilitate optimal dye positioning. Indeed, according to CD data, the duplex part of TBA25 may be partially unfolded, resulting in a slightly destabilized G4 with single-stranded flanks, and could explain different T_m_ values obtained upon monitoring of melting by UV- and CD-spectroscopy. In contrast, interactions with elongated version TBA63 led to a sharp decrease in the fluorescence signal comparable to that of single module TBA15 with the exception of the dye **3** (Table 2). The same dependence was observed when the dyes were mixed with the duplex ON1-ON2. These results comply with those obtained previously for the system dapoxyl dye/DAP-10 aptamer [19] and crystal violet – duplex-G4 system [24] and could be explained by the greater rigidity of the two-module structure, which does not allow dyes to occupy the position optimal for increased fluorescence light-up. Moreover, in some cases, alternative dye binding to the duplex module that does not increase the fluorescence signal can be an issue.

### 3.3. Interaction of GFP Chromorophore Analogues with Ribo-Variants of TBA

The common change in the topology of G-quadruplexes from antiparallel to parallel while switching from deoxyribo- to ribo-series may allow us to study the effect of a different G4 topology on fluorescence intensity enhancement but could also lead to changes in the structure of the inter-module junction. We synthesized and characterized RNA analogues of TBA15 and TBA31: ON15 and ON31, respectively. CD spectra of these ONs confirmed parallel G4 folding (Figure 2D) – a common feature of RNA G-quadruplexes with the notable exception of GFP-like RNA aptamer [33]. CD-melting at 265 nm (the parallel G4-specific maximum) gave monophasic curves, and similar results were obtained upon Abs.-monitoring (Figure 2E). Thermal stabilities increased from ON15 to ON31, supporting the positive effects of the duplex modules in the series (Figure 2E, Table 1). The binding of **BO** with ribo-analogues led to a fluorescence intensity enhancement (Table S1), comparable to those previously obtained for the deoxyribo-variants [32]. These results indirectly confirm that ON31 structure also contains a junction, which can enhance the light-up effect upon dye binding.

All dyes listed in Table 2 showed decreased sensitivity to the presence of an additional duplex module. Among them, derivative 6 exhibited about 20-fold fluorescence increase upon complexation with ON31 equal to deoxy-variant, reflecting the lack of sensitivity to the G4 topology for the two-module variant. Analogue 2 presented the same 6-fold increase in the fluorescence signal upon binding with both ON15 and ON31, presumably indicating a different binding mode of the dye. Surprisingly, derivatives 9–11 exhibited some ”light-up” effect in complex with ON31, but not with TBA31 (Figure 3, Table S1). Thus we found several leader compounds that demonstrated selective fluorescence intensity enhancement in complex with two-module (duplex+G4) structures in which a G4 part has parallel or antiparallel topology.

### 3.4. Interaction of GFP Chromophore Analogues with Alternative G4 Structures (LTR-III and Tel23a) Containing Additional Stabilizing Elements

To confirm the general nature of dye fluorescence enhancement in G4-duplex dual structures, we studied changes in the fluorescence intensity of GFP analogues upon binding to other G4 structures with adjusting structural modules – LTR-III and human telomeric sequence Tel23a. NMR studies of LTR-III, G-rich sequence located in the U3 promoter region of the HIV-1 long terminal repeat (LTR), revealed the formation of a unique quadruplex–duplex hybrid consisting of a three-layer (3 + 1) G-quadruplex scaffold and a 12-nt diagonal loop containing a conserved duplex-stem [34]. Besides the duplex part, which could allow binding of the dye between G4 and duplex modules, other elements may also contribute to constraining the dye in a planar conformation that increases the quantum yield of fluorescence. Indeed, X-ray structures of Spinach and Mango III aptamers show an important role of a non-canonical base trio in the dye accommodation [33,35,36]. In this study, we also used human telomeric sequence Tel23a that predominantly forms (3 + 1) G-quadruplex with T:A:T triple base structure that caps the bottom G-tetrad (hybrid 2) in potassium buffers [37]. CD spectra of LTR-III and Tel23a in our study are consistent with the previously reported ones [34,38]. This data confirms the secondary structures that are schematically shown in Figure 2D: a hybrid (3+1) G4 (Tel23a) and a hybrid G4 with an intra-loop duplex module (LTR-III). Melting curves of Tel23a and LTR-III were monophasic (Figure 2E) both by CD and UV absorbance spectroscopy at 265/295 nm (Table 1).

Then we studied the increase of fluorescence for all the dyes upon binding to LTR-III (Table S1). In most cases, the increase of fluorescence was lower in comparison to TBA31 and its analogues. However, “light-up pattern” for most of the compounds (Table 2 and Table S1) was similar to that for TBA analogues, with the exception of compounds 12–17 (Figure 3). Note that derivatives 4 and 14-17 shared pyridinyl moiety that apparently is responsible for the effect and could be used as a starting point for further optimization of dye - LTR-III pairs. As far as the structure of Tel23a joint is not stabilized by the duplex module, all the dyes from Table 2 poorly lighted up in comparison to TBA analogues. This data supports our hypothesis of the contribution of duplex modules to enhanced fluorescence of GFP chromophore analogues upon binding to various G4 structures.

## 4. Conclusions

Here we studied the influence of varied additional duplex modules on the fluorescence increase for synthetic GFP chromophore analogues upon binding to two-module (duplex+G4) structures. Screening of a large number of dyes and varying the duplex module length gave several dyes that light up by more than 20 times when binding to the two-module TBA aptamers and their RNA analogues and fluoresce in the blue to orange region of the visible spectrum. Duplex modules of moderate length enhance fluorescence, while long ones should be avoided. The negative effect of the long duplex module can be attributed to the increased rigidity of the two-module structure that prevents optimal positioning of the dye in the inter-module cavity. In spite of general positive input from the duplex module, the cavity at the duplex-quadruplex interface differs for various known structures. Thus, some optimization of the primary, secondary and tertiary structure for novel structures will be beneficial for increased light-up of dyes. The data obtained in our study will be helpful for the design of improved two-module signaling elements used in aptamer-based biosensors.

## Figures and Tables

**Table 1:** Oligonucleotide sequences and melting temperatures.

Code	Sequence (5′→3′)	T_m_ °C
TBA15	GGTTGGTGTGGTTGG	53 ± 1
TBA25	TGGTAGGTTGGTGTGGTTGGGGCCA	45 ± 2; 65 ± 2 ^a^
TBA31	CACTGGTAGGTTGGTGTGGTTGGGGCCAGTG	54 ± 3; 66 ± 1 ^a^
TBA63	ATAAAATAAAAAATTACACTGGTAGGTTGGTGTGGTTGGGGCCAGTGTAATTTTTTATTTTAT	57 ± 3; 67 ± 1 ^a^
ON1	ATAAAATAAAAAATTACACTGG	57 ± 1
ON2	CCAGTGTAATTTTTTATTTTAT	
ON15	r(GGUUGGUGUGGUUGG)	47 ± 2
ON31	r(CACUGGUAGGUUGGUGUGGUUGGGGCCAGUG)	63 ± 1
LTR-III	GGGAGGCGTGGCCTGGGCGGGACTGGGG	71 ± 2
Tel23a	AGGGTTAGGGTTAGGGTTAGGGT	69 ± 1

^a^—biphasic melting.

**Table 2:** Fluorescence intensity enhancement of selected GFP chromophore analogues upon binding to oligonucleotides.

Compound #	Fluorescence Intensity Enhancement								
	TBA31	TBA 15	TBA25	TBA63	ON1+2	ON31	ON15	LTR-III	Tel23a
**1a**	7.9 ± 0.08	2.2 ± 0.09	15.9 ± 0.24	2.3 ± 0.07	1.1 ± 0.08	2.7 ± 0.05	1.7 ± 0.03	3.0 ± 0.08	2.5 ± 0.05
**1b**	7.4 ± 0.08	2.0 ± 0.08	11.8 ± 0.20	2.9 ± 0.12	1.2 ± 0.06	2.7 ± 0.12	1.7 ± 0.08	5.2 ± 0.06	3.5 ± 0.10
**1c**	7.5 ± 0.16	2.3 ± 0.04	14.9 ± 0.21	2.8 ± 0.09	1.1 ± 0.04	3.2 ± 0.10	1.8 ± 0.06	5.1 ± 0.14	3.4 ± 0.08
**1d**	9.9 ± 0.12	2.5 ± 0.03	23.0 ± 0.25	3.2 ± 0.03	1.1 ± 0.07	3.1 ± 0.10	2.4 ± 0.09	3.9 ± 0.08	3.7 ± 0.14
**1e**	12.3 ± 0.13	2.6 ± 0.05	20.6 ± 0.29	2.8 ± 0.10	1.1 ± 0.05	3.6 ± 0.07	2.0 ± 0.05	8.1 ± 0.11	4.7 ± 0.07
**1f**	7.9 ± 0.17	2.0 ± 0.09	8.8 ± 0.18	1.8 ± 0.07	1.1 ± 0.08	2.5 ± 0.03	1.5 ± 0.09	4.2 ± 0.10	2.8 ± 0.02
**2**	10.1 ± 0.16	2.4 ± 0.02	8.7 ± 0.17	1.4 ± 0.05	1.5 ± 0.02	6.2 ± 0.12	6.1 ± 0.10	3.4 ± 0.13	2.6 ± 0.05
**3**	18.8 ± 0.28	2.1 ± 0.12	19.1 ± 0.21	12.1 ± 0.15	4.0 ± 0.09	6.2 ± 0.14	2.1 ± 0.03	3.4 ± 0.12	12.4 ± 0.19
**4**	16.5 ± 0.18	3.3 ± 0.13	11.4 ± 0.18	4.2 ± 0.13	1.2 ± 0.03	6.8 ± 0.13	2.6 ± 0.09	14.9 ± 0.20	6.2 ± 0.12
**5a**	12.4 ± 0.20	3.3 ± 0.06	10.1 ± 0.10	5.6 ± 0.12	1.3 ± 0.04	3.6 ± 0.07	1.9 ± 0.07	2.7 ± 0.10	2.0 ± 0.02
**5b**	12.1 ± 0.15	3.0 ± 0.06	10.6 ± 0.13	2.0 ± 0.07	1.1 ± 0.04	3.3 ± 0.03	1.4 ± 0.11	2.3 ± 0.06	2.7 ± 0.01
**6**	20.5 ± 0.22	2.9 ± 0.11	31.7 ± 0.40	4.8 ± 0.13	1.9 ± 0.02	19.0 ± 0.25	7.1 ± 0.09	4.9 ± 0.09	7.1 ± 0.16
**7**	8.2 ± 0.09	2.0 ± 0.10	14.3 ± 0.20	2.3 ± 0.09	1.1 ± 0.06	4.6 ± 0.13	2.1 ± 0.03	2.9 ± 0.05	2.1 ± 0.05
**8**	9.2 ± 0.12	2.3 ± 0.06	18.1 ± 0.18	2.4 ± 0.05	1.2 ± 0.04	11.0 ± 0.13	4.7 ± 0.11	1.8 ± 0.07	3.5 ± 0.07

#—Fluorescence intensity enhancement (mean of three replicates (±SD)) was defined as the ratio of fluorescence intensity of fluorophore+oligonucleotide solution to the fluorescence intensity of free fluorophore solution (5 µM both in 20 mM Tris-HCl, pH 7.8, 100 mM KCl).

## Supplementary Materials

The following are available online at https://www.mdpi.com/1424-8220/20/3/915/s1, Table S1: Fluorophores from the library, their optical properties and the results of interaction with deoxyribo- (TBA31, TBA15 and LTR-III) and ribo- (ON31 and ON15) oligonucleotides, Figure S1: Fluorescence spectra of bound (in buffer) and free (in water) leader fluorophores.
