# Supplementary material for: Short Duplex Module Coupled to G-Quadruplexes Increases Fluorescence of Synthetic GFP Chromophore Analogues
Source: Sensors (Basel). 2020 Feb 9;20(3):915. doi: 10.3390/s20030915 (PMC7038953; doi:10.3390/s20030915)
Supplement: Supplementary file 1 [file sensors-20-00915-s001.pdf]

Supplementary Materials:

Short Duplex Module Coupled to G-Quadruplexes Increases Fluorescence of Synthetic GFP Chromophore Analogues

Table S1. Chromophores from the library, their optical properties and the results of interaction with deoxyribo- (**TBA31**, **TBA15** and **LTR-III**) and ribo- (**ON31** and **ON15**) oligonucleotides.

code	Structure	Abs[a]	Em[a]	Maximal emission enhancement					Synthesis
				TBA31	TBA15	ON31	ON15	LTR-III	
**BO**	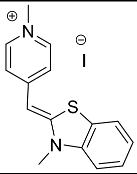	440	485	2708	34	1240	260	54	[1]
**1a (A1)**[b]	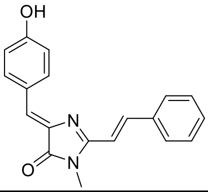	426	526 (545)[c]	7.9±0.08	2.2±0.09	2.7±0.05	1.7±0.03	3.0±0.08	[2]
**1b (A7)**	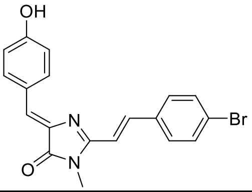	438	532 (550)	7.4±0.08	2.0±0.08	2.7±0.12	1.7±0.08	5.2±0.06	[3]
**1c (A15)**	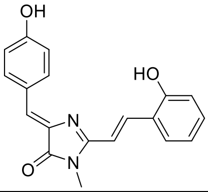	428	535 (552)	7.5±0.16	2.3±0.04	3.2±0.10	1.8±0.06	5.1±0.14	[3]
**1d (A18)**	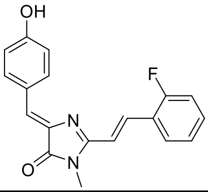	427	530 (550)	9.9±0.12	2.5±0.03	3.1±0.10	2.4±0.09	3.9±0.08	[3]
**1e (A22)**	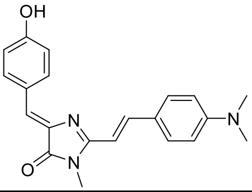	486	595 (616)	12.3±0.13	2.6±0.05	3.6±0.07	2.0±0.05	8.1±0.11	[3]
**1f (N873)**	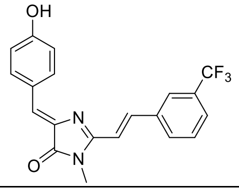	430	548 (555)	7.9±0.17	2.0±0.09	2.5±0.03	1.5±0.09	4.2±0.10	[3]

**2 (M2372a)**	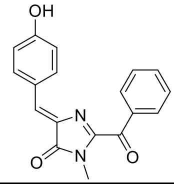	425	590 (565)	10.1±0.16	2.4±0.02	6.2±0.12	6.1±0.10	3.4±0.13	[3]
**3 (N154)**	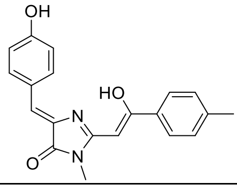	433	505 (500)	18.8±0.28	2.1±0.12	6.2±0.14	2.1±0.03	3.4±0.12	[4]
**4 (N908)**	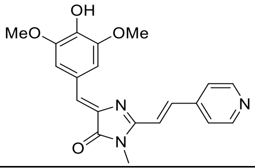	449	600 (605)	16.5±0.18	3.3±0.13	6.8±0.13	2.6±0.09	14.9±0.20	[3]
**5a (SA158)**	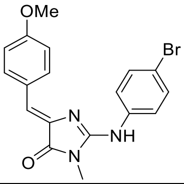	377	428 (455)	12.4±0.20	3.3±0.06	3.6±0.07	1.9±0.07	2.7±0.10	[5]
**5b (M1944b)**	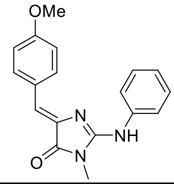	376	460 (450)	12.1±0.15	3.0±0.06	3.3±0.03	1.4±0.11	2.3±0.06	[5]
**6 (M1975)**	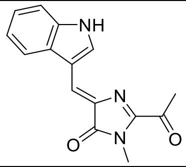	473	565 (535)	20.5±0.22	2.9±0.11	19.0±0.25	7.1±0.09	4.9±0.09	[6]
**7 (ZS292)**	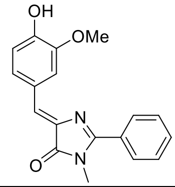	402	495 (505)	8.2±0.09	2.0±0.10	4.6±0.13	2.1±0.03	2.9±0.05	[7]
**8 (M2385b)**	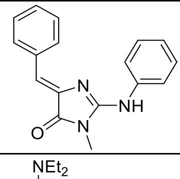	368	432 (473)	9.2±0.12	2.3±0.06	11.0±0.13	4.7±0.11	1.8±0.07	[5]
**9 (M 2371a)**	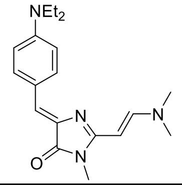	498	592	1.5	1.1	6.2	1.5	6.2	[3]
**10(N 848.4)**	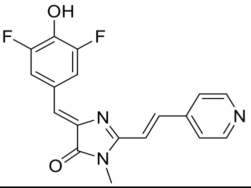	430	570	2.0	1.4	5.7	1.6	2.7	[3]

**11(M 2368c)**	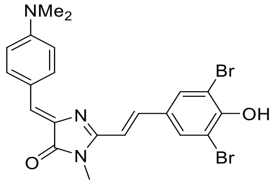	526	620	2.6	1.5	6.1	1.6	3.7	[3]
**A 2**	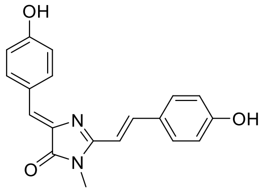	437	548	4.7	1.6	2.9	1.7	5.3	[2]
**A 4**	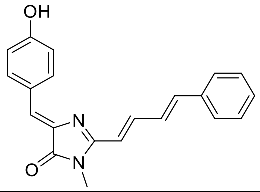	447	575	2.3	2.3	1.9	1.3	2.8	[3]
**A 8**	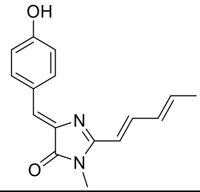	414	520	5.7	3.4	3.1	2.4	2.2	[8]
**A 9**	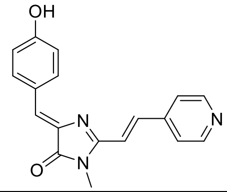	430	545	3.4	1.3	2.3	1.6	2.8	[3]
**A 11**	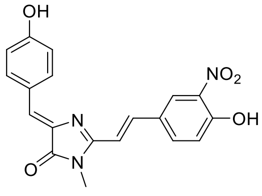	410	560	1.2	1.2	2.0	1.7	1.5	[3]
**A 12**	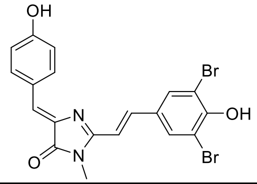	472	550	3.8	1.2	1.9	1.1	3.1	[3]
**A 16**	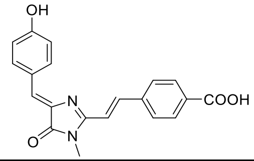	430	540	1.8	1.4	1.7	1.9	1.8	[3]
**A 19**	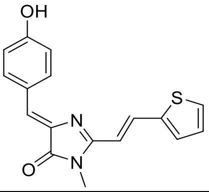	438	563	2.1	1.3	4.3	1.3	2.6	[9]

**A 20**	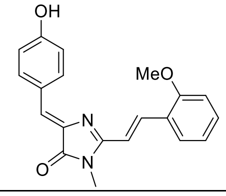	434	530	4.8	3.8	2.3	1.4	3.7	[3]
**E 7**	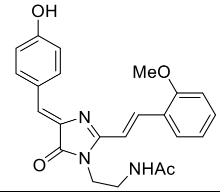	432	530	2.7	1.5	2.2	1.5	3.3	[3]
**E 4**	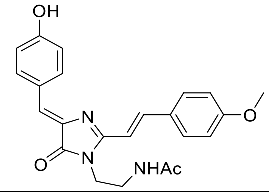	435	533	1.9	1.4	1.7	1.3	4.0	[3]
**M 1971c**	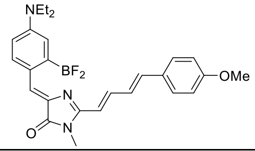	608	742	3.7	1.1	1.9	1.5	1.9	[10]
**M 2371c**	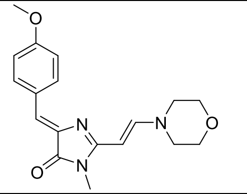	435	510	1.6	1.1	2.1	1.5	1.8	[3]
**M 2371b**	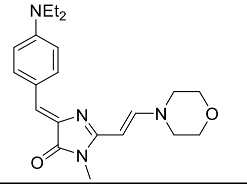	496	580	1.5	1.1	2.8	1.7	4.2	[3]
**M 2372b**	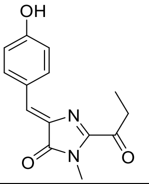	419	546	1.3	1.1	2.0	2.1	1.1	[3]
**M 800**	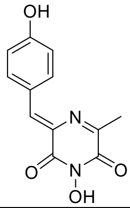	406	530	1.3	1.2	4.0	3.5	1.3	[11]
**M 790**	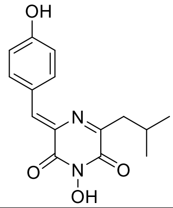	410	533	1.4	1.2	2.1	2.0	1.3	[11]

**M 802**	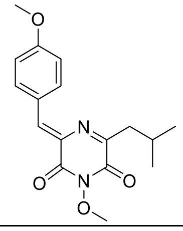	415	514	1.4	1.5	1.8	1.6	1.1	[11]
**GA 01**	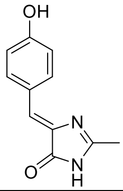	372	460	1.5	1.2	1.9	1.4	1.1	[12]
**GA 02**	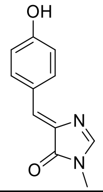	365	456	1.5	1.2	2.7	1.7	1.1	[12]
**GA 03**	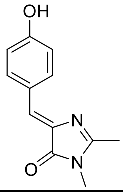	390	450	1.6	1.2	2.0	1.8	1.6	[13]
**GA 04**	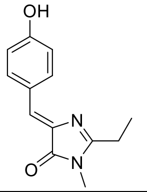	369	455	1.3	1.4	1.9	2.0	2.0	[14]
**12 (GA 05)**	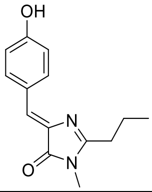	370	456	1.3	1.2	1.9	1.7	11.1	[12]
**GA 06**	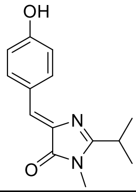	370	452	1.5	1.3	1.9	1.9	1.2	[12]
**GA 12**	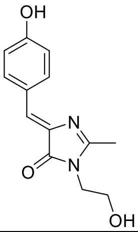	370	453	1.6	1.2	1.8	1.7	1.3	[15]
**GA 16**	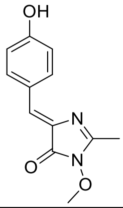	370	457	1.6	1.2	1.9	1.3	1.1	[11]

**GA 18**	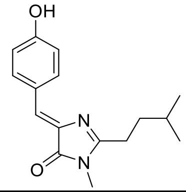	369	458	1.6	1.2	2.0	1.5	2.0	[11]
**SA 198**	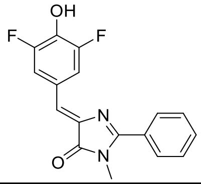	386	448	1.2	1.2	2.0	1.4	1.0	[7]
**SA 49**	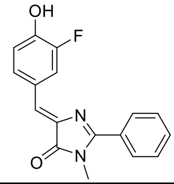	392	493	1.1	1.2	1.4	1.1	0.9	[7]
**M 2296**	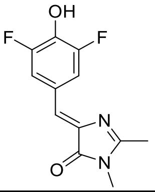	363	470	1.0	1.1	1.6	1.2	1.1	[16]
**M 2227**	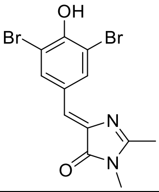	365	–[d]	1.3	1.1	1.6	1.5	1.2	[16]
**GC 05**	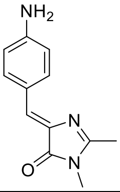	392	487	1.5	1.2	1.7	1.2	1.1	[17]
**GC 04**	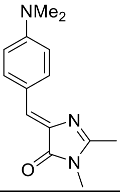	442	523	1.6	1.2	1.4	1.2	2.3	[17]
**GC 01**	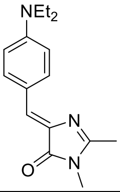	453	526	1.4	1.2	3.7	1.2	1.1	[18]
**GC 03**	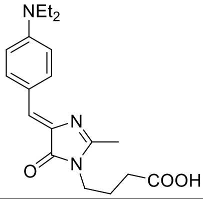	457	528	1.3	1.1	1.4	1.3	1.1	[19]

**N 641**	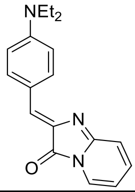	564	601	5.0	1.4	2.4	1.2	7.2	[20]
**13 (M 1933)**	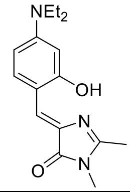	464	515	1.8	1.2	1.4	1.2	10.7	[19]
**M 739**	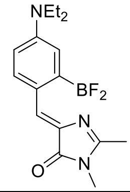	520	563	1.1	1.1	1.3	1.0	1.3	[17]
**MKA 67**	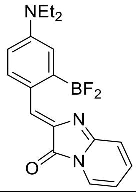	603	626	1.1	1.0	-	-	1.2	[20]
**M 2360**	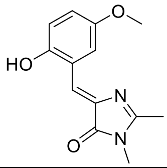	400	574	1.6	1.2	1.4	1.3	1.3	[21]
**ZS 62**	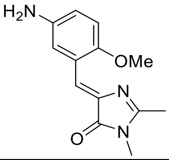	348	476	1.2	1.1	2.0	1.5	1.2	[3]
**GC 07**	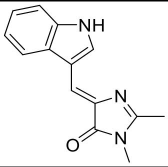	404	473	1.8	1.4	1.5	1.5	1.4	[18]
**ZS 289a**	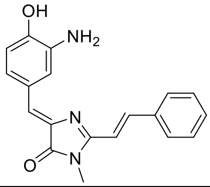	448	–[d]	3.7	1.9	2.3	1.8	2.7	[7]
**ZS 291**	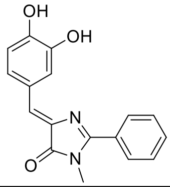	401	500	2.9	1.4	2.2	1.6	1.8	[7]
**ZS 295**	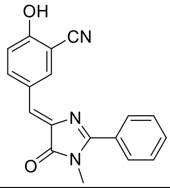	382	475	2.1	1.4	2.1	1.3	1.5	[7]

**ZS 297**	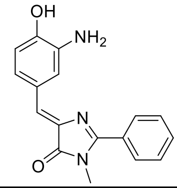	412	–[d]	2.1	1.5	2.1	1.3	1.7	[7]
**14 (ZS 285a)**	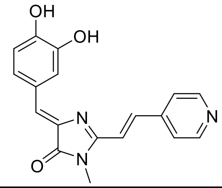	446	575	3.5	1.3	2.4	1.5	9.5	[7]
**ZS 286**	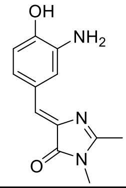	388	–[d]	1.5	1.3	1.6	1.2	1.6	[7]
**ZS 298**	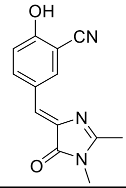	371	485	1.7	1.2	1.6	1.2	1.1	[7]
**ZS 299b**	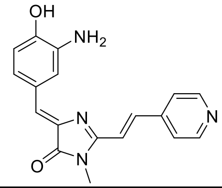	450	–[d]	2.2	1.2	1.8	1.8	1.6	[7]
**ZS 300a**	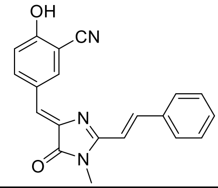	426	535	5.7	3.7	2.7	1.7	6.2	[7]
**M 2491**	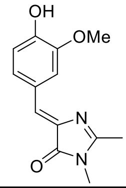	378	471	1.3	1.2	1.5	1.3	1.2	[7]
**M 2499a**	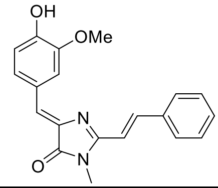	370	465	1.7	1.2	1.5	1.4	1.2	[7]
**M1583.7**	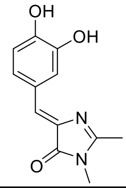	379	475	1.3	1.2	2.3	1.4	1.4	[7]
**N 906**	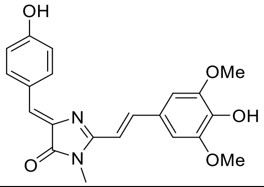	430	556	2.8	1.1	2.0	1.5	6.4	[3]

**N 860c**	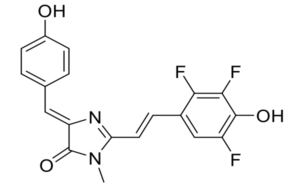	461	537	2.6	1.4	1.7	1.7	4.1	[3]
**N 860b**	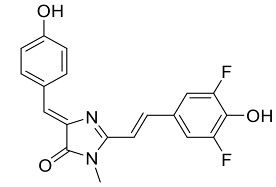	450	550	2.9	1.3	1.8	1.5	3.7	[3]
**N 914**	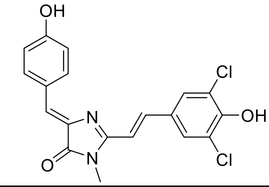	473	550	2.1	1.1	1.5	1.4	2.9	[3]
**N 886**	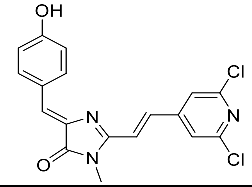	438	565	4.2	1.6	2.9	2.1	2.3	[3]
**N 871b**	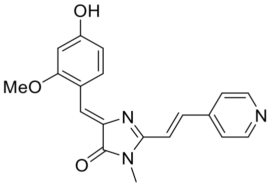	455	570	3.7	1.6	2.5	2.7	7.1	[3]
**15 (N 901)**	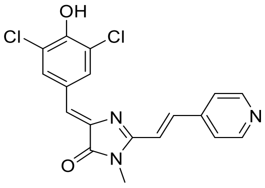	430	–[d]	3.3	1.7	2.8	4.1	8.8	[3]
**N 858**	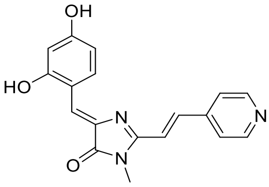	424	560	1.3	1.3	1.6	1.6	1.4	[3]
**N 848.3**	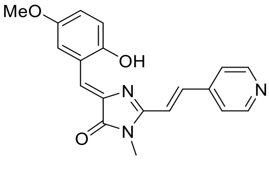	450	531	4.4	1.8	3.1	2.1	2.6	[3]

**M 2368d**	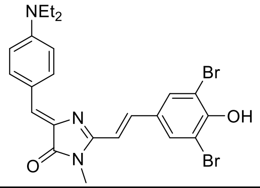	530	620	1.7	1.2	2.5	1.6	3.7	[3]
**16 (N 848.1)**	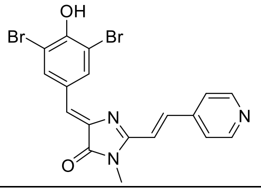	436	550	2.6	1.3	2.9	2.1	8.9	[3]
**17 (N 863)**	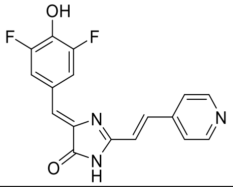	424	590	2.0	1.4	3.0	1.2	7.7	[3]

[a] – maxima position in nm; [b] - the designation used in Figure S1 is shown in parentheses; [c] – maximum emission positions in complex with **TBA31** is shown in brackets; [d] – weak emission.

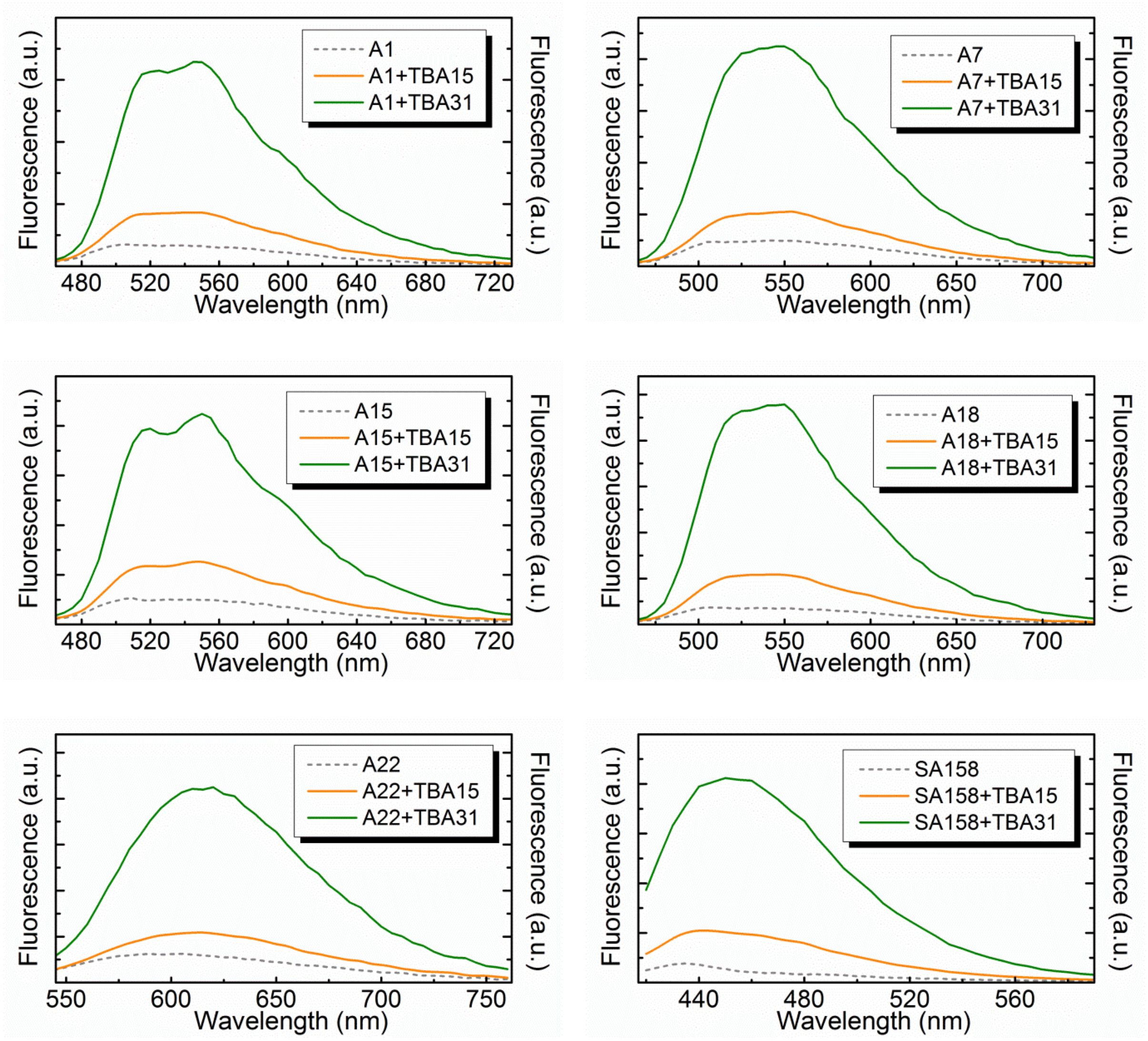

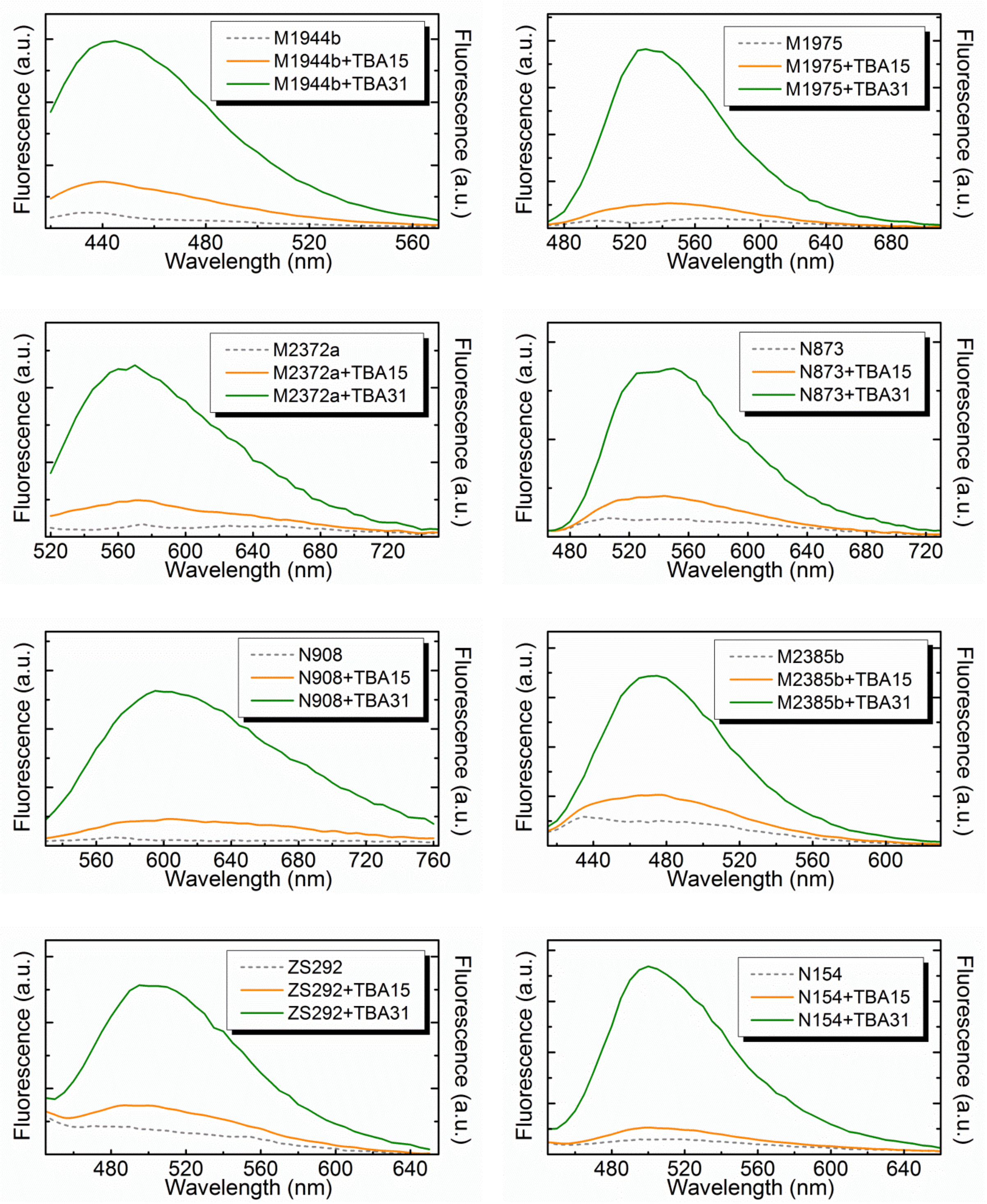

Figure S1. Fluorescence spectra of bound (in buffer) and free (in water) leader fluorophores.

References

1. Turaev, A.V.; Tsvetkov, V.B.; Tankevich, M.V.; Smirnov, I.P.; Aralov, A.V.; Pozmogova, G.E.; Varizhuk, A.M. Benzothiazole-based cyanines as fluorescent "light-up" probes for duplex and quadruplex DNA. *Biochimie* **2019**, *162*, 216–228, doi:10.1016/j.biochi.2019.04.018.
2. Yampolsky, I.V.; Kislukhin, A.A.; Amatov, T.T.; Shcherbo, D.; Potapov, V.K.; Lukyanov, S.; Lukyanov, K.A. Synthesis and properties of the red chromophore of the green-to-red photoconvertible

fluorescent protein Kaede and its analogs. *Bioorg. Chem.* **2008**, *36*, 96–104, doi:10.1016/j.bioorg.2007.12.003.

3. Povarova, N.V.; Zaitseva, S.O.; Baleeva, N.S.; Smirnov, A.Y.; Myasnyanko, I.N.; Zagudaylova, M.B.; Bozhanova, N.G.; Gorbachev, D.A.; Malyshevskaya, K.K.; Gavrikov, A.S., et al. Red-Shifted Substrates for FAST Fluorogen-Activating Protein Based on the GFP-Like Chromophores. *Chem.Eur. J.* **2019**, *25*, 9592–9596, doi:10.1002/chem.201901151.
4. Golodukhina, S.V.; Baleeva, N.S.; Mineyev, K.S.; Baranov, M.S. Reversible condensation of 4-arylidene-1,2-dimethyl-1H-imidazol-5(4H)-ones with aromatic acyl chlorides. *Chem. Heterocycl. Compd.***2015**, *51*, 944–947, doi:10.1007/s10593-015-1802-6.
5. Smirnov, A.Y.; Baleeva, N.S.; Zaitseva, S.O.; Mineev, K.S.; Baranov, M.S. Derivatives of Azidocinnamic Acid in the Synthesis of 2-Amino-4-Arylidene-1H-Imidazol-5(4H)-Ones. *Chem. Heterocycl. Compd.* **2018**, *54*, 625–629, doi:10.1007/s10593-018-2318-7.
6. Bozhanova, N.G.; Baranov, M.S.; Sarkisyan, K.S.; Gritcenko, R.; Mineev, K.S.; Golodukhina, S.V.; Baleeva, N.S.; Lukyanov, K.A.; Mishin, A.S. Yellow and Orange Fluorescent Proteins with Tryptophan-based Chromophores. *ACS Chem. Biol.* **2017**, *12*, 1867–1873, doi:10.1021/acschembio.7b00337.
7. Chen, C.; Baranov, M.S.; Zhu, L.D.; Baleeva, N.S.; Smirnov, A.Y.; Zaitseva, S.O.; Yampolsky, I.V.; Solntsev, K.M.; Fang, C. Designing redder and brighter fluorophores by synergistic tuning of ground and excited states. *Chem. Commun.* **2019**, *55*, 2537–2540, doi:10.1039/c8cc10007a.
8. He, X.; Bell, A.F.; Tonge, P.J. Synthesis and spectroscopic studies of model red fluorescent protein chromophores. *Org. Lett.* **2002**, *4*, 1523–1526, doi:10.1021/ol0200403.
9. Chuang, W.T.; Chen, B.S.; Chen, K.Y.; Hsieh, C.C.; Chou, P.T. Fluorescent protein red Kaede chromophore; one-step, high-yield synthesis and potential application for solar cells. *Chem. Commun.* **2009**, 6982–6984, doi:10.1039/b908542d.
10. Bozhanova, N.G.; Baranov, M.S.; Baleeva, N.S.; Gavrikov, A.S.; Mishin, A.S. Red-Shifted Aminated Derivatives of GFP Chromophore for Live-Cell Protein Labeling with Lipocalins. *Int. J. Mol. Sci.* **2018**, *19*, 3778, doi:10.3390/Ijms19123778.
11. Baranov, M.S.; Fedyakina, I.T.; Shchelkanov, M.Y.; Yampolsky, I.V. Ring-expanding rearrangement of 2-acyl-5-arylidene-3,5-dihydro-4H-imidazol-4-ones in synthesis of flutimide analogs. *Tetrahedron* **2014**, *70*, 3714–3719, doi:10.1016/j.tet.2014.04.013.
12. Baranov, M.S.; Solntsev, K.M.; Lukyanov, K.A.; Yampolsky, I.V. A synthetic approach to GFP chromophore analogs from 3-azidocinnamates. Role of methyl rotors in chromophore photophysics. *Chem. Commun.* **2013**, *49*, 5778–5780, doi:10.1039/c3cc41948g.
13. Kojima, S.; Ohkawa, H.; Hirano, T.; Maki, S.; Niwa, H.; Ohashi, M.; Inouye, S.; Tsuji, F.I. Fluorescent properties of model chromophores of tyrosine-66 substituted mutants of Aequorea green fluorescent protein (GFP). *Tetrahedron Lett.* **1998**, *39*, 5239–5242, doi: 10.1016/S0040-4039(98)01031-4.
14. Yampolsky, I.V.; Balashova, T.A.; Lukyanov, K.A. Synthesis and Spectral and Chemical Properties of the Yellow Fluorescent Protein zFP538 Chromophore. *Biochemistry* **2009**, *48*, 8077–8082, doi:10.1021/bi900719x.
15. Petersen, M.A.; Riber, P.; Andersen, L.H.; Nielsen, M.B. Synthesis and characterization of model compounds for the neutral green fluorescent protein chromophore. *Synthesis* **2007**, 3635–3638, doi:10.1055/s-2007-990852.
16. Song, W.J.; Strack, R.L.; Svensen, N.; Jaffrey, S.R. Plug-and-Play Fluorophores Extend the Spectral Properties of Spinach. *J. Am. Chem. Soc.* **2014**, *136*, 1198–1201, doi:10.1021/ja410819x.
17. Baranov, M.S.; Solntsev, K.M.; Baleeva, N.S.; Mishin, A.S.; Lukyanov, S.A.; Lukyanov, K.A.; Yampolsky, I.V. Red-Shifted Fluorescent Aminated Derivatives of a Conformationally Locked GFP Chromophore. *Chem.-Eur. J.* **2014**, *20*, 13234–13241, doi:10.1002/chem.201403678.
18. Baldridge, A.; Kowalik, J.; Tolbert, L.M. Efficient Synthesis of New 4-Arylideneimidazolin-5-ones Related to the GFP Chromophore by 2+3 Cyclocondensation of Arylideneimines with Imidate Ylides. *Synthesis* **2010**, 2424–2436, doi:10.1055/s-0029-1218796.
19. Baldridge, A.; Feng, S.H.; Chang, Y.T.; Tolbert, L.M. Recapture of GFP Chromophore Fluorescence in a Protein Host. *Acs Comb. Sci.* **2011**, *13*, 214–217, doi:10.1021/co200025e.

20. Baleeva, N.S.; Myannik, K.A.; Yampolsky, I.V.; Baranov, M.S. Bioinspired Fluorescent Dyes Based on a Conformationally Locked Chromophore of the Fluorescent Protein Kaede. *Eur. J. Org. Chem.* **2015**, 5716–5721, doi:10.1002/ejoc.201500721.
21. Chuang, W.T.; Hsieh, C.C.; Lai, C.H.; Lai, C.H.; Shih, C.W.; Chen, K.Y.; Hung, W.Y.; Hsu, Y.H.; Chou, P.T. Excited-State Intramolecular Proton Transfer Molecules Bearing o-Hydroxy Analogues of Green Fluorescent Protein Chromophore. *J. Org. Chem.* **2011**, *76*, 8189–8202, doi:10.1021/jo2012384.
